# Genome-wide analysis of chromatin packing in *Arabidopsis thaliana* at single-gene resolution

**DOI:** 10.1101/gr.204032.116

**Published:** 2016-08

**Authors:** Chang Liu, Congmao Wang, George Wang, Claude Becker, Maricris Zaidem, Detlef Weigel

**Affiliations:** 1 Department of Molecular Biology, Max Planck Institute for Developmental Biology, 72076 Tübingen, Germany;; 2 Center for Plant Molecular Biology (ZMBP), University of Tübingen, 72076 Tübingen, Germany;; 3 Institute of Digital Agriculture, Zhejiang Academy of Agriculture Sciences, Hangzhou 310029, China

## Abstract

The three-dimensional packing of the genome plays an important role in regulating gene expression. We have used Hi-C, a genome-wide chromatin conformation capture (3C) method, to analyze *Arabidopsis thaliana* chromosomes dissected into subkilobase segments, which is required for gene-level resolution in this species with a gene-dense genome. We found that the repressive H3K27me3 histone mark is overrepresented in the promoter regions of genes that are in conformational linkage over long distances. In line with the globally dispersed distribution of RNA polymerase II in *A. thaliana* nuclear space, actively transcribed genes do not show a strong tendency to associate with each other. In general, there are often contacts between 5′ and 3′ ends of genes, forming local chromatin loops. Such self-loop structures of genes are more likely to occur in more highly expressed genes, although they can also be found in silent genes. Silent genes with local chromatin loops are highly enriched for the histone variant H3.3 at their 5′ and 3′ ends but depleted of repressive marks such as heterochromatic histone modifications and DNA methylation in flanking regions. Our results suggest that, different from animals, a major theme of genome folding in *A. thaliana* is the formation of structural units that correspond to gene bodies.

The spatial organization of the genome in the nucleus is critical for many cellular processes (Van Bortle and Corces 2012). It has been broadly accepted that the packing of chromatin inside the nucleus is not random but structured at several hierarchical levels (Gibcus and Dekker 2013). Cytological studies have indicated that each chromosome occupies a distinct domain within the nucleus, termed chromosome territory, which is stable during the interphase of the cell cycle.

Microscopy-based methods have limited power to reveal fine-grained chromatin structures at the kilobase level and therefore have recently been complemented by PCR and sequencing-based methods. The chromosome conformation capture (3C) approach, which targets specific loci, has been widely used to examine juxtaposition between specific transcription units and remote enhancer elements (Dekker et al. 2002). A more generic approach, the Hi-C technique, has been developed and first applied to human cells to detect nuclear interactions throughout the genome (Lieberman-Aiden et al. 2009). These and other Hi-C experiments revealed topologically associating domains (TADs), which are local packing units separated by boundaries that are enriched for binding of CTCF insulators and highly expressed genes, as prevailing structural features of metazoan genomes (Dixon et al. 2012; Dekker et al. 2013).

In contrast to animals, TADs are not prominent in the genome of *Arabidopsis thaliana*, a possible explanation being the absence of canonical insulator proteins in this species and in other plants (Wang et al. 2015). Instead, the global Hi-C picture is dominated by strong intra- and inter-chromosomal interactions between interactive heterochromatic islands (IHIs) (Feng et al. 2014), which form a structure that has also been called KNOT engaged element (KEE) (Grob et al. 2014). These regions are typically 20 to 150 kb long and may anchor the megabase-sized chromatin loops visible under the microscope (Fransz et al. 2002). At higher resolution, over 1000 TAD-boundary-like and insulator-like regions have been identified, which correlate with open chromatin and highly transcribed genes (Wang et al. 2015). In addition, a special structural feature named “positive strips” was described in this Hi-C map with 2-kb resolution. Positive strips showed frequent interactions with neighboring chromatin and were enriched with H3K27me3, a histone modification associated with Polycomb repressive complexes (PRCs).

Many chromatin packing studies have focused on structural details at the level of individual gene bodies, such as recent Hi-C maps from human cells (Jin et al. 2013; Rao et al. 2014; Ma et al. 2015; Mifsud et al. 2015). Through increasing sequencing depth, either throughout the genome (Jin et al. 2013; Rao et al. 2014) or in selectively enriched regions of interests (Ma et al. 2015; Mifsud et al. 2015), these studies have interrogated chromatin structure at high resolution. This has led to systematic discoveries of interactions between genes and their regulatory elements in space, which were linked with chromatin architectural proteins (e.g., CTCF) and histone modifications. In contrast, Hi-C studies with *A. thaliana* have focused on chromatin structure at a scale of 2 to 20 kb (Feng et al. 2014; Grob et al. 2014; Wang et al. 2015), which often exceeds the size of individual gene bodies in this species, making it difficult to address questions concerning interactions between genes and their regulatory elements. Here, we present an analysis of *A. thaliana* chromatin interaction patterns at the gene level resolution, focusing on the systematic identification of small chromatin loops. Our results suggest that gene bodies in *A. thaliana* largely outline local chromatin packing patterns and provide a framework in which transcriptional regulation can be investigated in the context of three-dimensional space.

## Results and discussion

### Identification of chromatin loops

The *A. thaliana* genome is crowded, with over 33,000 genes in 135 Mb (The Arabidopsis Genome Initiative 2000). Gene bodies and intergenic regions are, on average, around 2 to 3 kb. Reporter gene analyses have shown that in many cases a gene's expression pattern can be reproduced by a 2- to 3-kb promoter sequence, implying that the majority of the *cis*-regulatory elements in *A. thaliana* are located close to their target genes (Lee et al. 2006). If chromatin loops mediate interactions between, e.g., enhancers and promoters, they would likely be at a scale of a few kb. In order to identify such small chromatin loops, chromatin must be fragmented into pieces shorter than the loop size, using DNase I (Ma et al. 2015), micrococcal nuclease (Hsieh et al. 2015), or frequently cutting restriction enzymes. We have previously used a four-cutter enzyme to generate a Hi-C map (Wang et al. 2015), in which we observed that at short distances, the contact frequency between two loci on the same chromosome showed a power-law decay with genomic distance (Supplemental Fig. 1). Such power-law behavior, which reflects the physical property and packing patterns of chromatin in nuclear space, has been typically found for chromatin interactions over genomic distances at the Mb scale, suggesting that it is feasible to adapt previously established methods (Jin et al. 2013; Ay et al. 2014) to model and identify small chromatin loops in *A. thaliana*.

We developed a pipeline to identify statistically significant patterns indicative of small chromatin loops (see details in Methods). For every chromatin interaction observed in our data set, we estimated its expected sequencing counts based on a spline-fitting approach (Ay et al. 2014). All chromatin interactions could be fitted well with a negative binomial distribution (Supplemental Fig. 2), which we used to determine the statistical significance of each pair of chromatin interactions. Biases that affected the count number of each interaction that appeared in our sequencing reads, such as fragment length, PCR efficiency, mappability, and restriction site density of the corresponding chromatin segments, were taken into account (Supplemental Fig. 3). Because sequencing depth is a major limiting factor for the number of chromatin loops that can be confidently identified (Jin et al. 2013), we added two newly produced Hi-C replicates to our published *A. thaliana* Hi-C reads (Wang et al. 2015). For each of the four data sets, the majority, 61%–79%, of identified chromatin loops could be called from the combined pool of the other three data sets with relaxed *q* values (Supplemental Fig. 4). With stringent read mapping and filtering, we retained over 162 million Hi-C reads from our combined data set, of which around 125 million reads were from intra-chromosomal interactions (Supplemental Table 1). In total, we identified over 20,000 chromatin loops from the euchromatic chromosome arms (*q* < 0.01) (Supplemental Table 2).

**Figure 1.**
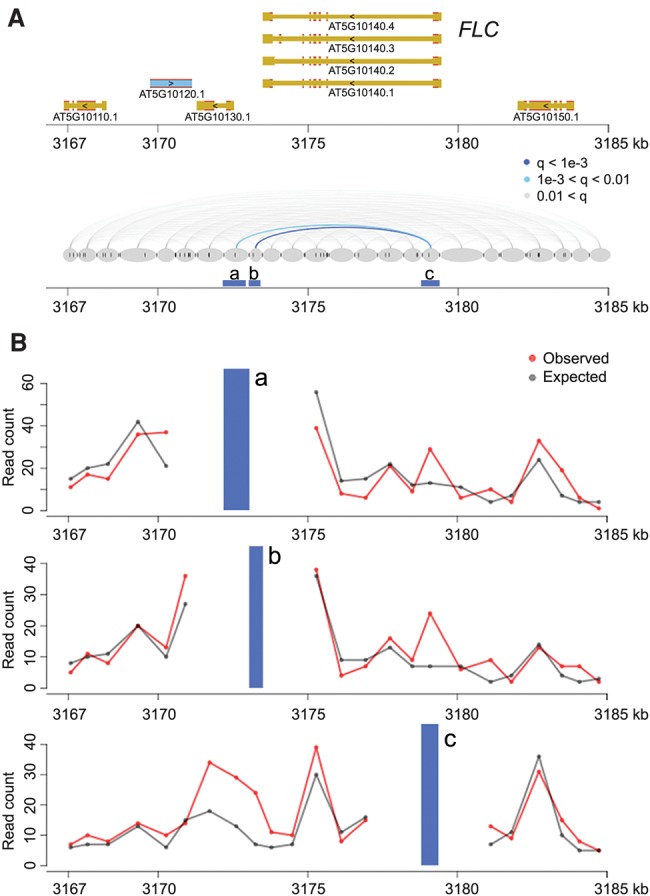
Chromatin loops at *FLC*. (*A*) The *FLC* locus including 5 kb up- and downstream was divided into segments (gray ovals) based on the location of restriction sites (dark gray vertical ticks between and inside ovals). Chromatin interactions (with distances over 2 kb) within this region are shown in the *bottom* diagram as lines connecting the corresponding segments and colored according to the *q*-values. Segments a, b, and c are analyzed in detail in *B*. (*B*) Observed and expected read counts with three segments. The blue vertical bars correspond to the anchor regions highlighted in *A*.

Among the identified chromatin loops was one at the *FLOWERING LOCUS C* (*FLC*) locus (Fig. 1). A 3C experiment had already shown that an ∼2.7-kb region covering the promoter and the transcription start site of *FLC* makes contact with sequences immediately downstream from the gene (Crevillen et al. 2013). Our Hi-C data confirmed this chromatin loop and further suggested that the strongest interaction occurred between the *FLC* 3′ flanking region and the first exon (Fig. 1), which is highly enriched for histone variant H2A.Z and a transcription-activating mark (H3K9ac) (Jegu et al. 2014). With a relaxed *q*-value cutoff (*q* < 0.05), we re-identified another known loop, between the *AT2G34655* (also known as *APOLO*) promoter and the *PINOID* (*PID*) coding region (Supplemental Fig. 5; Ariel et al. 2014). In an earlier study, the *APOLO* promoter had been found to contact the *PID* promoter when *PID* expression was suppressed by N-1-naphthylphthalamic acid (NPA), a chemical that blocks auxin transport (Ariel et al. 2014). *PID* RNA was expressed in our samples, and we did not detect chromatin looping between the *APOLO* and *PID* promoters, which is consistent with the finding that opening of this loop structure occurs simultaneously with activation of *PID* expression (Ariel et al. 2014). The detection of a significant contact between the *APOLO* promoter and *PID* coding region in our material suggests that the interaction between *APOLO* promoter and *PID* depends on *PID* expression.

We did not detect contact signals at two other loci with known chromatin loops, *TFL1* and *FT*, both of which have rather restricted spatial and/or circadian expression patterns (Liu et al. 2013, 2014; Cao et al. 2014). Like almost all whole-genome studies in plants, the output of our Hi-C experiment reflects the average pattern in all cells collected. Our samples comprised mostly leaves, which in turn consist mostly of mesophyll, with other cell types, such as meristematic, epidermal, vasculature, guard, and trichome cells, each contributing much less to the overall signal. The Hi-C patterns we describe thus are likely representative either for those shared among different vegetative shoot cell types and/or for patterns from mesophyll cells.

### H3K27me3 is enriched for long-range and promoter-promoter interactions

We next asked whether loop formation correlates with functional annotation of gene parts or epigenetic features of the involved sequences. By separating chromatin segments into gene bodies, gene peripheries, or intergenic, we found that gene bodies were overrepresented among chromatin loops (Fisher's exact test, *P* < 2.2 × 10^−16^) (Supplemental Fig. 3C). We also examined whether chromatin loops were enriched for specific chromatin states that are reflected in correlated epigenetic marks. From a nine-chromatin-state annotation, we found that chromatin loops larger than 6 kb tended to avoid state 4, which corresponded to intergenic or distal promoter regions with substantial H3K27me3 levels (Supplemental Fig. 6A; Sequeira-Mendes et al. 2014), while a six-chromatin-state annotation indicated that these chromatin loops tended to involve state 2, also characterized by H3K27me3 (Supplemental Fig. 6B; Wang et al. 2015). This observation suggested that H3K27me3 or correlated marks in gene bodies might be enriched in large (≥6 kb) loop structures.

**Figure 2.**
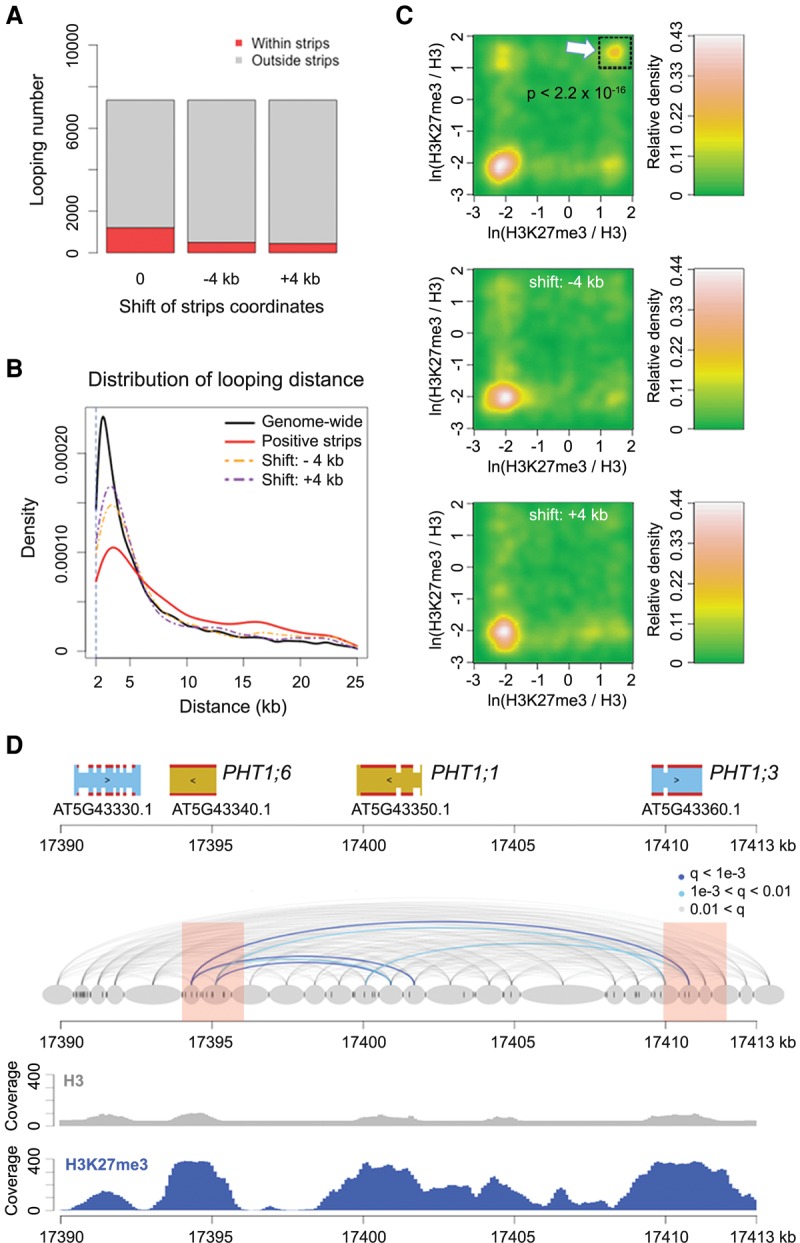
H3K27me3 and chromatin loops. (*A*) Proportion of chromatin loops related to “positive strips,” defined as regions with strong Hi-C interaction with neighboring chromatin (Wang et al. 2015). Loops shorter than 6 kb are not included. “−4k b” and “+4 kb” represent controls in which the coordinates of annotated positive strips were shifted 4 kb up- or downstream. (*B*) Chromatin loop sizes in regions overlapping with positive strips, compared with genome-wide background and shifted coordinates as in *A*. (*C*) Density of H3K27me3 on chromatin loops that have at least one interacting partner in positive strip regions. For each pair of interacting chromatin segments, the H3K27me3 signal on both segments was calculated as the natural base logarithm of the ratio between normalized H3K27me3 and H3 coverage. The distribution of these pairwise values is shown as a matrix of relative density, generated with the “bkde2D” function in the R software package “KernSmooth” (grid size = 80, bandwidth = 0.15). (*Middle* and *bottom*) Coordinates of positive strips are shifted as in *A*. The *P*-value comes from testing for enrichment of loops in the highlighted square relative to a permutation-based null distribution of background, estimated via shifting positive strip coordinates ±4 kb and ±8 kb. (*D*) Chromatin loops in a region with three *PHT1* genes. Positive strip regions are highlighted in pink. Normalized H3 and H3K27me3 ChIP-seq coverages are shown *below*. See Figure 1A for additional annotation.

Regions that had higher contact strength with neighboring chromatin, which we have called positive strips (Wang et al. 2015), were enriched for long-range chromatin loops (one-sided Wilcoxon-Mann-Whitney test, *P* = 5.5 × 10^−15^ between the red and orange lines, and *P* = 2.2 × 10^−24^ between the red and purple lines in Fig. 2B) (Fig. 2A,B). Among all identified chromatin loops larger than 6 kb, a subset of those with at least one chromatin segment overlapping with positive strips had strong H3K27me3 signals on both interacting partners (Fig. 2C). Figure 2D shows one such example, in which several chromatin loops connect three closely related genes encoding phosphate transporters (*PHT1;1*, *PHT1;3*, and *PHT1;6*) that are not expressed in leaves (Mudge et al. 2002). All three were strongly enriched for H3K27me3, and two overlapped with positive strip regions.

**Figure 3.**
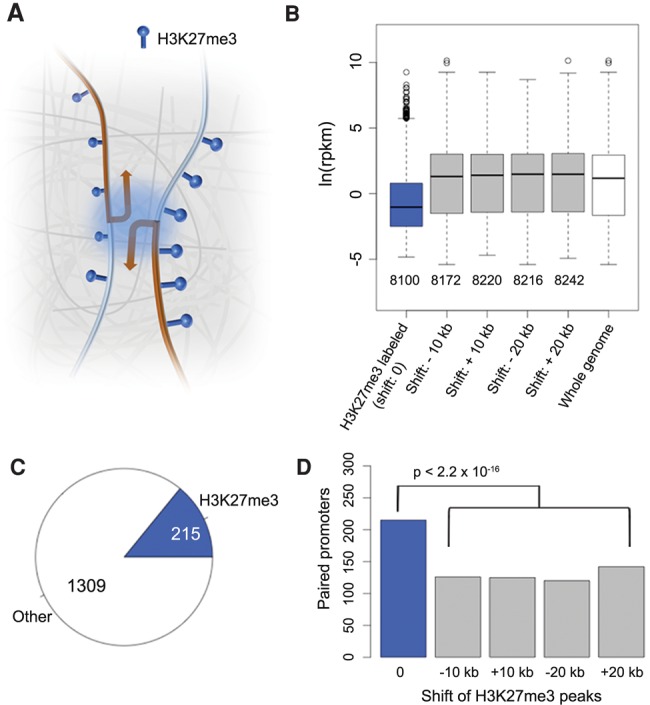
H3K27me3 and promoter-promoter interactions. (*A*) Cartoon of interactions between promoters, which are enriched for the H3K27me3 mark. We tested promoter-promoter interactions for overrepresentation of H3K27me3 on both promoters, including convergent, divergent, and tandem promoter pairs. (*B*) Correlation between H3K27me3 and gene expression. In the four control sets, upon shifting the coordinates of H3K27me3 peaks, genes marked with H3K27me3 are no longer associated with reduced expression. (*C*) Fraction of promoters enriched with H3K27me3 relative to all promoter-promoter interactions. Loops shorter than 6 kb were not considered. (*D*) Correlation between H3K27me3 and promoter-promoter interactions. Shifted controls as in *B*. *P*-values indicate significance relative to the permutation-based null distribution of the background.

These results prompted us to ask whether in *A. thaliana* certain H3K27me3-marked loci were clustered in three-dimensional space to form a “repressive chromatin hub,” similar to Polycomb-targeted *HOX* clusters in animals (Fig. 3A; Bantignies and Cavalli 2011; Schoenfelder et al. 2015). From our ChIP-seq data, we extracted 8100 genes where at least 30% of their promoters (defined as ±500-bp regions flanking the TSSs [transcription start sites]) overlapped with H3K27me3 peaks (Fig. 3B). For the control sets, we extracted a similar number of genes (Fig. 3B) according to a shifted H3K27me3 landscape rather than a random permutation, because the distribution of this histone mark was not uniform across the genome. After shifting the coordinates of H3K27me3 peaks either 10 or 20 kb upstream or downstream, the “H3K27me3” label was no longer correlated with suppressed gene expression (all control sets had *P*-values ≥0.99 from one-sided Wilcoxon-Mann-Whitney tests against genome-wide RNA expression levels) (Fig. 3B).

From our chromatin loop list, we found 1,524 pairs of interacting promoters, with 215 (14%) having H3K27me3 marks on both promoters (Fig. 3C; Supplemental Table 3). The control sets had substantially fewer pairs (Fig. 3D). In conclusion, for promoter-promoter interactions, H3K27me3 presence at both promoters is more common than expected by chance, even though the majority of promoter-promoter interactions do not require the dual H3K27me3 mark. Specific capture of H3K27me3-labeled chromatin loops (Fullwood and Ruan 2009) will be a useful approach to determine whether interactions between H3K27me3-marked promoters can be seen over longer genomic distances and to what extent such physical interactions are linked with transcriptional regulation.

Physical interaction between H3K27me3-marked, allelic *FLC* loci is induced by cold exposure (Rosa et al. 2013), but this no longer occurs in mutants deficient in H3K27me3 deposition on *FLC* chromatin (De Lucia et al. 2008). At a chromosomal scale, strong interactions have been reported within certain H3K27me3-marked regions, which were dramatically reduced in PRC2 (Polycomb repressive complex 2) mutants (Feng et al. 2014). Despite the association of H3K27me3, PRC2 and clustering, it remains uncertain whether H3K27me3 deposition is required for chromatin clustering. LHP1 (LIKE HETEROCHROMATIN PROTEIN 1) protein is almost exclusively associated with H3K27me3-marked chromatin (Turck et al. 2007; Zhang et al. 2007), and it distributes as speckles in nuclear space (Nakahigashi et al. 2005), which possibly reflects the structural clustering of H3K27me3-marked loci. However, LHP1 is not involved in depositing H3K27me3 (Turck et al. 2007), and *FLC* clustering is not impaired in *lhp1* mutants (Rosa et al. 2013), implying that is not essential for chromatin clustering.

In animals, structural proteins, such as CTCF and the structural maintenance of chromosomes (SMC) complexes, which include cohesin and condensin, play critical roles in organizing chromatin structure (Jeppsson et al. 2014; Zlotorynski 2015). Although plants do not have CTCF proteins, a high-resolution microscopy analysis of *A. thaliana* cohesin subunit SMC3 (AT2G27170) and condensin subunit CAP-D3 (AT4G15890) has revealed that these proteins can form nonoverlapping speckles throughout the euchromatic nucleoplasm of interphase nuclei, suggesting their involvement in organizing interphase chromatin (Schubert et al. 2013). It will be of interest to determine whether plant SMCs affect promoter-promoter interactions or local chromatin loops in general.

### Transcribed genes do not show strong preference to couple

**Figure 4.**
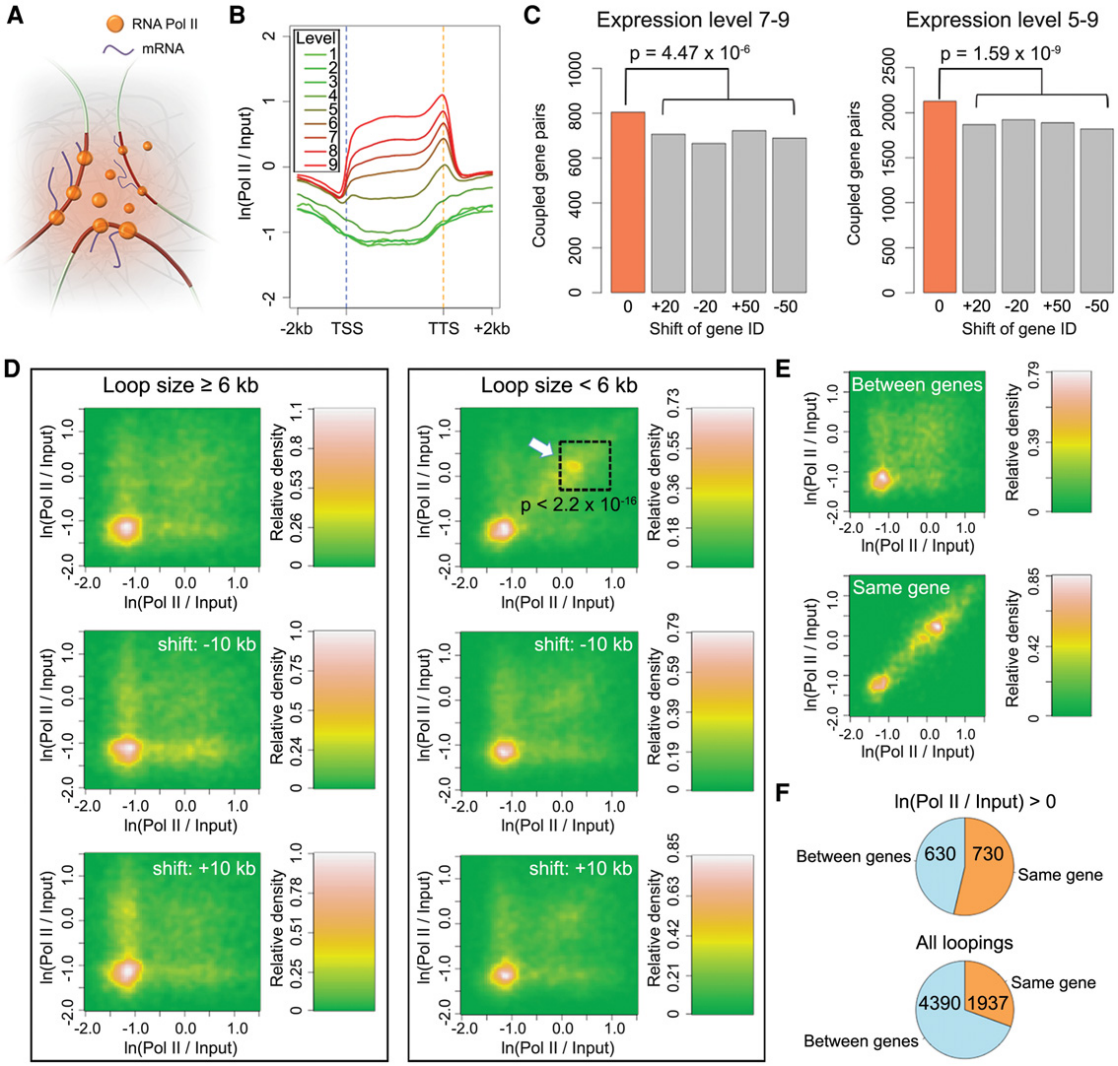
Coupling of expressed genes. (*A*) A cartoon of the transcription factory model. (*B*) Correlation between RNA Pol II binding and gene expression levels. Genes are scaled to align their TSSs and TTSs. (*C*) Coupled gene pairs that are actively expressed. The background (gray) was estimated by reassigning new expression levels to genes according to the values from the 20th or 50th genes upstream or downstream. Loops shorter than 6 kb were not considered. *P*-values indicate significance relative to the permutation-based null distribution of background. (*D*,*E*) Association of RNA Pol II with chromatin loops. The *lower* two plots in *D* always show controls where the coordinates of RNA Pol II signals (measured as the natural base logarithm of the ratio between normalized Pol II and input coverage) were shifted 10 kb up- or downstream. The *P*-value comes from testing for enrichment of loops in the highlighted square relative to a permutation-based null distribution of background, estimated by shifting Pol II signal coordinates ±10 kb and ±20 kb. The two panels in *E* only include loops shorter than 6 kb. See Figure 2C legend for more information. (*F*) Interactions between and within genes that are connected by chromatin loops with interacting partners that overlap gene bodies. The numbers indicate pairs of genes in each category. In both comparisons, only loops shorter than 6 kb were considered, and for RNA Pol II on *top*, only loops where both interacting partners were enriched for Pol II (having a signal/input ratio larger than 1) were selected.

The term “transcription factory” has been used to describe nuclear structures that consist of concentrated RNA polymerases. These are thought to facilitate efficient transcription of multiple colocalized genes (Sutherland and Bickmore 2009). This model has been refined using advanced microscopy techniques. While the majority of active RNA Pol II molecules are globally dispersed in both animal and plant nuclei, a small subset forms clusters (Schubert 2014; Zhao et al. 2014; Schubert and Weisshart 2015). To ask whether highly expressed genes that are at least 6 kb apart are more likely to interact with each other than less highly expressed genes, we selected genes based on their transcript levels, which correlated with Pol II binding (Fig. 4B). A first group contained highly expressed genes (levels 7–9), while the second contained a broader range of expressed genes (levels 5–9). Compared to the control data set, both groups were slightly but significantly enriched for gene pairs forming chromatin loops between their transcribed regions (Fig. 4C; Supplemental Table 4). This observation suggests that in *A. thaliana*, transcribed genes have only a weak tendency to cluster in three-dimensional space. Chromosomal-scale compartmentalization of chromatin has been shown in human cells, where active and inactive chromatin reside in different domains (“AB” compartment) (Lieberman-Aiden et al. 2009). A similar arrangement in *A. thaliana* has been deduced from their Hi-C maps (Grob et al. 2014). Highly transcribed genes showed a tendency to couple in our data, suggesting that there is “AB” type compartmentalization at a local level, in domains of a few tens of kb.

On the other hand, when we examined chromatin loops smaller than 6 kb, we found that a subset of them had strong Pol II binding on both interacting partners (Fig. 4D, highlighted with an arrow), perhaps reflecting active RNA polymerases aggregated within short distances (Schubert and Weisshart 2015). To address whether this pattern arises from interactions between highly expressed genes located close to each other in the genome and/or interactions within the same gene body, we focused on Pol II ChIP-seq peaks in small chromatin loops. We found that the dual presence of Pol II at both interaction partners was preferentially associated with interactions within the same gene body (Fig. 4E). Among all small-sized chromatin loops where both interacting partners mapped to gene bodies, 31% (1937/6327) of gene-gene interactions were restricted to one gene; this proportion was 54% (730/1360) for loops having strong Pol II binding (Fig. 4F). Taken together, our results suggest that highly expressed *A. thaliana* genes do not predominately couple with each other over long distances. At short distances, interactions between chromatin strongly bound by Pol II mainly occur within genes.

### Self-loops around genes are common

**Figure 5.**
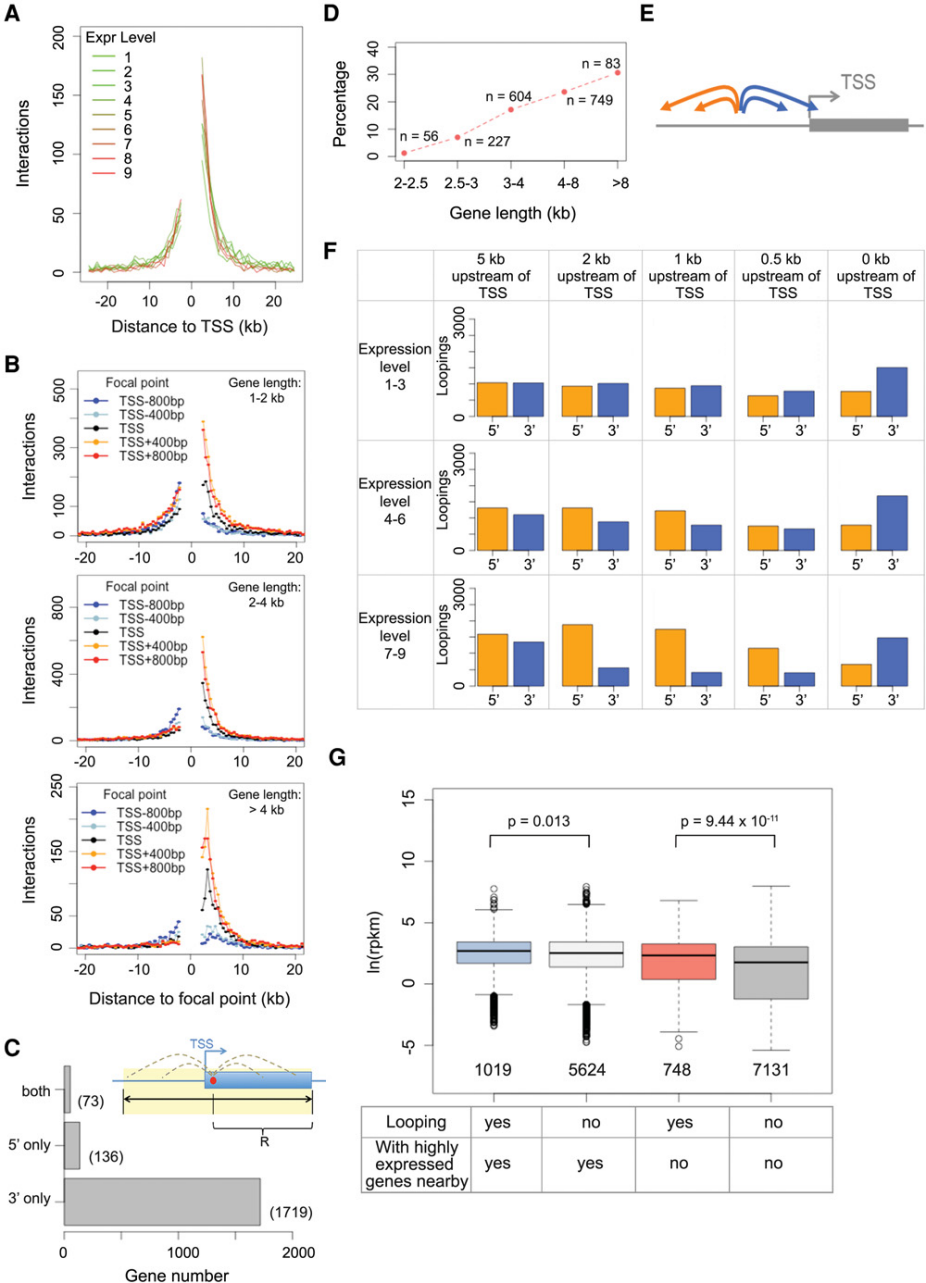
Detailed characterization of gene loops. (*A*) Chromatin loop sizes and direction around TSSs grouped according to gene expression level. (*B*) Chromatin loop sizes and direction from various anchor points around TSSs, as a function of gene length. (*C*) Identification of genes having self-looped structures. For each gene, the search radius “R” was based on the distance between the focal point (red dot) and TTS. With this strategy, genes with chromatin loops are classified as “3′ only,” “5′ only,” or “both,” depending on the locations of the interacting partners. (*D*) Fraction of genes with self-looped structures classified as “3′ only” in *C*. (*E*,*F*) Patterns of chromatin loops related to regions upstream of genes. For a focal point located upstream of a TSS, its corresponding chromatin segment can have interacting partners located at 3′ or 5′ regions with respect to the gene (*E*). A series of focal points (5, 2, 1, 0.5, and 0 kb upstream of TSSs) were analyzed on all genes in the genome divided by expression levels (*F*). (*G*) Correlation between gene loop and expression, given as the natural base logarithm of RPKM (reads per kilobase per million reads). Two pairs of comparisons on transcriptional activities are shown, depending on whether there is a highly expressed gene (level 7–9) located within 5 kb of genes of interest. The *P*-values indicate Wilcoxon-Mann-Whitney test results.

Because our Pol II analysis revealed that many highly expressed genes formed chromatin loops (Fig. 4E), we examined the exact patterns of chromatin loops on gene bodies. We found that regardless of gene expression or length, TSSs preferentially formed chromatin loops with regions located downstream with respect to direction of transcription (Fig. 5A,B). On the other hand, chromatin regions containing TTSs (transcription termination sites) preferentially looped with upstream regions (Supplemental Fig. 7). This pattern was not specific to the boundaries of transcribed sequences, as we found similar patterns when analyzing gene body regions a few hundred bp inward from TSSs or TTSs (Fig. 5B; Supplemental Fig. 7), suggesting that at a local level, many *A. thaliana* genes adopted self-loops.

In total, 1792 genes had self-loops between the 5′ and 3′ portions of their transcribed regions (Supplemental Table 5), corresponding to 12% of the 14,672 candidate genes, for which our algorithms could potentially identify gene loops (see details in Methods; Fig. 5C). When dividing genes by length, we found that the fraction of genes having their 5′ transcribed regions specifically involved in forming self-loop structures was higher for those with longer gene bodies (Fig. 5D). We named these 1792 self-loops “gene loops,” although they did not necessarily form between TSSs and TTSs; in fact, these interactions were reminiscent of the gene “crumples” or globules discovered in a recent high-resolution Hi-C map from yeast (Hsieh et al. 2015). It should be pointed out here that we likely underestimated the actual number of genes with loop structure because of our conservative cutoff for calling significant looping events. Moreover, it remains unresolved how many small genes have self-loops, as our study considered only chromatin loops larger than 2 kb.

### Gene loops are associated with higher expression of neighboring genes

Gene loops play various roles in modulating gene expression, such as maintaining transcriptional memory (Laine et al. 2009; Tan-Wong et al. 2009), suppressing bidirectional transcription (Tan-Wong et al. 2012), termination of transcription (Mukundan and Ansari 2013), and promoting intron-mediated enhancement of transcription (Moabbi et al. 2012). Several general factors related to transcription, such as a subunit of the RNA Pol II complex, Ssu72, and a TFIIB transcription factor, Sua7, contribute to the formation of gene loops in yeast (Singh and Hampsey 2007; Tan-Wong et al. 2012). Whether their plant homologs have similar functions remains unknown.

We found potential links between gene loops and transcriptional activity of neighboring chromatin regions. Compared to the background, genes neighboring regions with loops did not differ in terms of transcriptional direction or gene length but were expressed at higher levels (Supplemental Fig. 8). In parallel, on a genome-wide scale, we further examined the interaction directionality bias of regions close to gene bodies. The results suggest an “insulating effect” within a few kb upstream of highly expressed genes: Chromatin located within this range preferentially showed interaction with regions further upstream, but not downstream (Fig. 5E,F; Supplemental Fig. 9). This insulating effect was in line with our previous finding that highly expressed genes are enriched at insulator-like regions (Wang et al. 2015). Chromatin regions next to silent genes did not show any biased interaction directionality (Fig. 5F; Supplemental Fig. 9). The causality between the level of gene expression and the structure of its flanking chromatin regions is unclear, and some gene loop structures in *A. thaliana* may simply be the consequence of adjacent opened chromatin due to highly transcribed genes. If this is the case, for transgenes having strong promoters such as the cauliflower mosaic virus 35S promoter, the regions flanking insertion sites might acquire a new chromatin conformation that ultimately influences the expression of host genes adjacent to the transgene. Likewise, the presence of a highly expressed host gene next to a transgene insertion might contribute to variation in transgene activity.

### Silent genes with gene loops have unique epigenetic features

We assessed the connection between gene loops and expression levels. As mentioned above, because of possible influences from nearby highly expressed genes, we compared genes with self-loops that had at least one highly expressed gene within 5 kb and genes without such neighbors. In both groups, self-looping genes tended to have higher expression levels than non-self-looping genes (Fig. 5G). Upon grouping genes by length, we also observed these differences with genes that did not have highly expressed neighbors (Supplemental Fig. 10). These results suggest that gene loops in plants are positively associated with transcriptional activity.

Next, we asked whether genes with loop structure had unique genomic or epigenomic features that were in general associated with gene expression. We grouped genes according to expression levels and examined the distribution of seven histone modifications, five histone variants, DNA methylation, and DNase I hypersensitive sites over their transcribed regions (information from Stroud et al. 2012, 2014; Zhang et al. 2012; Luo et al. 2013; Yelagandula et al. 2014; Wang et al. 2015). For genes expressed at medium or high levels, we did not find obvious associations (Supplemental Fig. 11). Neither did classification by aspects of gene structure, such as exon/intron size, exon density, first exon length, and GC content, reveal differences (data not shown). Because looped genes tended to be expressed at higher levels (Fig. 5G), the transcription machinery per se, or certain general transcription factors, might be directly involved in forming gene loops. In yeast, inactivation of the “gene loop” factor Ssu72 reduces gene compaction not only at individual genes (Tan-Wong et al. 2012) but also on a global scale (Hsieh et al. 2015). An intriguing question to be addressed in the future is whether plant Ssu72 homologs (Hajheidari et al. 2013) are involved in gene loop formation.

**Figure 6.**
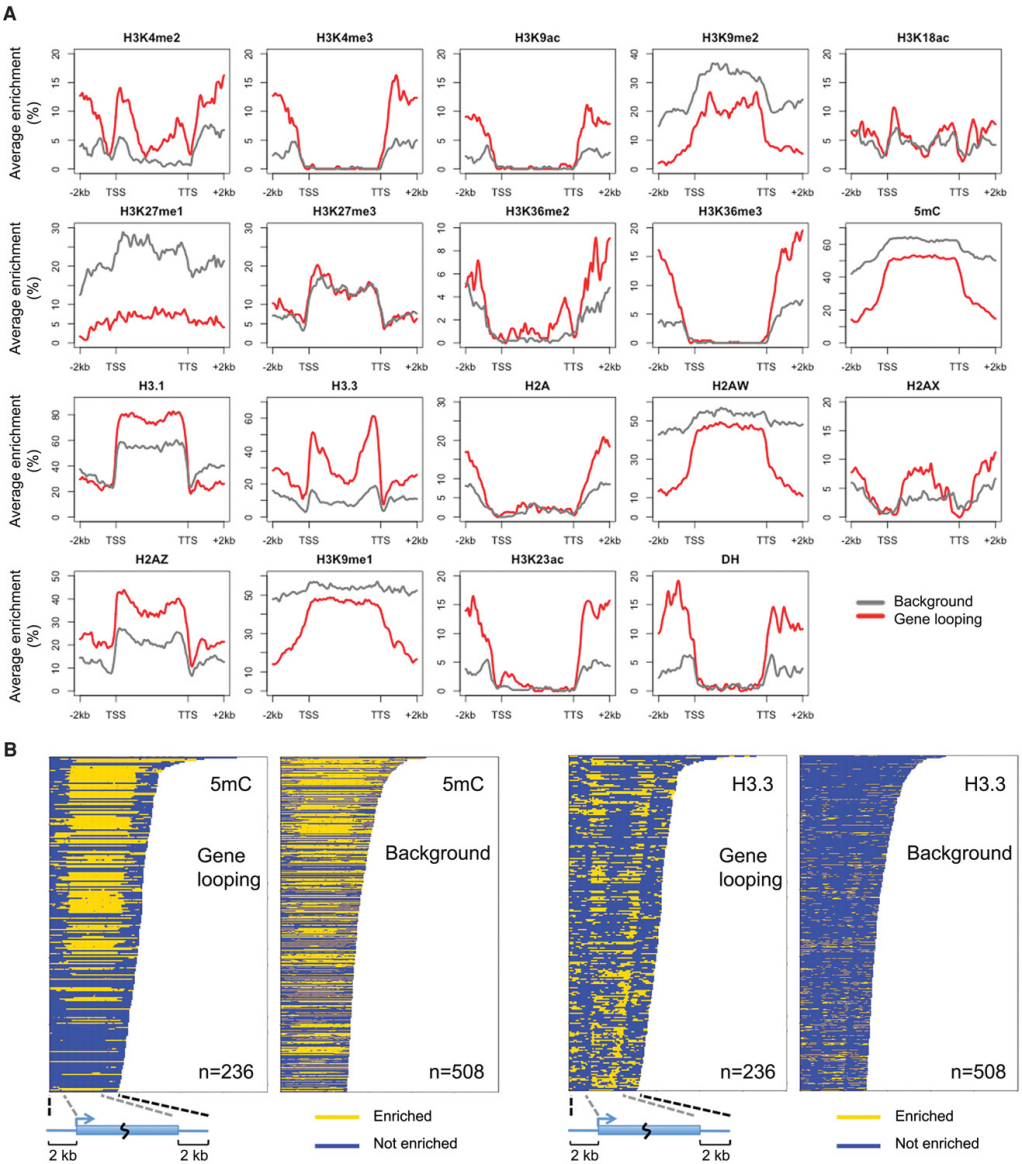
Epigenetic features associated with silent genes having gene loop conformation. (*A*) Epigenetic marks around genes with expression level 1–3. Genes are scaled to align their TSSs and TTSs. Average enrichment means the percentage of regions (calculated from 100-bp windows) enriched for the respective epigenetic mark. (*B*) Enrichment of DNA methylation and H3.3 on individual genes shown in *A*.

For genes which were silent or expressed at low levels (level 1–3), we found that gene loops were associated with enrichment for histone variant H3.3 in the gene body near TSSs and TTSs, and at the same time with depletion for DNA methylation and several heterochromatic marks in flanking regions (Fig. 6). The difference in DNA methylation in gene flanking regions was contributed by all three types of cytosine methylation (CG, CHG, and CHH) (Supplemental Fig. 12). Similar to animals, the *A. thaliana* H3 variant H3.3 is generally associated with transcribed genes, with H3.3 being preferentially enriched around TTSs (Stroud et al. 2012; Wollmann et al. 2012). Independently of expression levels, H3.3 is enriched at promoter regions of genes that tend to be responsive to environmental or developmental signals (Shu et al. 2014). In animals, H3.3 has been shown to play a role in gene silencing (Szenker et al. 2011), being required for the establishment of heterochromatin state on endogenous retroviral elements in embryonic stem cells (Elsasser et al. 2015). Because we had found that H3.3 was highly enriched at both ends of silent genes with a gene loop structure, we suspected that for these genes, gene loops might act in concert with the H3.3 depositing complex and thus help to confine the silencing effect to the target gene. It is also possible that gene loops reinforce gene silencing in cases where heterochromatin is limited to gene bodies. As can be seen from Figure 6, there was a sharp transition in heterochromatic marks such as DNA methylation, H3K9me1, H3K9me2, and H2A.W, between gene bodies of these silent genes and their flanking regions.

## Conclusions

Our analysis of *A. thaliana* Hi-C data has revealed important details of chromatin structure at very high resolution. Similar to the enrichment of interactions between H3K27me3-marked promoters, and interactions between H3K27me3-marked enhancers and their target sites that have been discovered in animals from high-resolution Hi-C data (Jin et al. 2013; Mifsud et al. 2015), the histone mark H3K27me3 is favored for *A. thaliana* genes with promoter-promoter interactions over long distances, suggesting a role of the Polycomb repressive complex in shaping genome organization. The distribution of the active form of RNA Pol II showed that in *A. thaliana* actively transcribed genes are not strongly coupled, even though gene loops might constitute a widespread phenomenon in *A. thaliana*. Gene loops are positively correlated with gene expression, suggesting a role in promoting transcription that is shared between plants and animals (Singh and Hampsey 2007). Gene loops also occur in silent genes or genes with low expression; the unique epigenetic profiles of these genes imply silencing mechanisms that are different from those of silent genes without gene loops. Considering the lack of classic TAD structures in *A. thaliana* (Feng et al. 2014; Grob et al. 2014; Wang et al. 2015), we conclude that the folding pattern of its genome differs from that of animals in that the preferential units of packing are gene bodies, similar to *S. cerevisiae* (Hsieh et al. 2015).

## Methods

### Plant material

*Arabidopsis thaliana* accession Columbia (Col-0) was grown at 23°C in long days (16 h light/8 h dark) on half-strength Murashige & Skoog (MS) medium supplemented with 1% sucrose and 0.3% Phytagel. The aerial portions of 10-d-old seedlings were harvested at Zeitgeber time (ZT) 6 h for Hi-C and ChIP-seq library preparation.

### Hi-C library preparation

Hi-C libraries were constructed as described (Wang et al. 2015) using DpnII as the restriction enzyme for chromatin fragmentation. For one round of preparation, nuclei extracted from 2 g fixed tissues were equally divided into five tubes and advanced in parallel. After biotin labeling of chromatin fragment ends and heat inactivation of DpnII, all five tubes were advanced to the ligation step. Subsequent chromatin DNA manipulations were performed as described (Wang et al. 2015). The final library molecules were sequenced on an Illumina HiSeq 2000 instrument with 2×101-bp reads.

### Hi-C read mapping and filtering

Read mapping, removal of PCR duplicates, and read filtering were performed as described (Wang et al. 2015) with a minor change: To minimize the trade-off between computing time and the amount of chimeric reads that could be successfully mapped, starting from the 5′ terminal 25 bp of each read, we performed 5-bp instead of 2-bp stepwise iterative mapping. Hi-C reads from each replicate used in this study are summarized in Supplemental Table 1.

### Calling chromatin loops

For chromatin digestion, we used a four-cutter restriction enzyme, DpnII, which produces mostly chromatin fragments below 500 bp (Wang et al. 2015). To define chromatin loci among which loops are called, we divided the genome into segments flanked by restriction sites. If applicable, neighboring fragments were merged, such that at the end the majority of segments had sizes ranging from 500 bp to 1 kb (Supplemental Fig. 3). During chromatin loop calling, the distance between two segments was approximated as the genomic distance between their centers. Similar to observations by Jin et al. (2013), we had previously found biases associated with Hi-C reads that mapped to loci separated by <1500 bp in the genome, which were mainly due to self-ligation products (Wang et al. 2015). In this study, we therefore set the lower boundary for mapping distance at 2 kb and called loops with statically significant contacts from segment pairs with intra-chromosomal distances between 2 and 25 kb.

In Hi-C experiments, in addition to chimeric reads coming from statistically significant chromatin loops, the following background reads are also expected: (1) reads from self-ligated fragments; (2) reads from random collisions of chromatin fragments during ligation; and (3) reads from stochastically formed chromatin loops, with interaction frequency largely dependent on physical distance. We focused on intra-chromosomal interactions between 2 and 25 kb. Among all filtered intra-chromosomal Hi-C reads, ∼25% fell in this interval, indicating that distance-dependent stochastic looping was a dominant factor. In this interval, we found that a log-linear relationship held between interaction frequency and intra-chromosomal distance (Supplemental Fig. 1).

All reads corresponding to contacts between segments having 2–25 kb distance were extracted from filtered Hi-C reads, except for reads that mapped to segments located in centromeric regions (Chr1, 13.7–15.9 Mb; Chr2, 2.45–5.50 Mb; Chr3, 11.3–14.3 Mb; Chr4, 1.80–5.15 Mb; Chr5, 11.0–13.35 Mb). A table describing these Hi-C reads counts is available in Supplemental Table 6. Next, a spline-fitting strategy (Ay et al. 2014) was employed to estimate the contact probability between two chromatin segments with a given genomic distance. The reads were assigned to their corresponding chromatin segments, sorted by interaction distance, and divided into 50 equal bins, so that each bin contained an equal number of reads. In this way, each bin contained interaction between segments having distance within [a, b], and the average per-bp contact count *C* for each bin was calculated as

(1) $$C_{i}=\frac{N_{i}}{b-a+1},$$

where *i* is the bin number, *N*_*i*_ is the total number of observed contacts in that bin, and *a* and *b* are the upper and lower bound of bin *i*, respectively. The average segment distance *D*_*i*_ was calculated as

(2) $$D_{i}=\frac{\sum_{j=a}^{b}\;(n_{j}\cdot j)}{\sum_{j=a}^{b}\;j},$$

where *n*_*j*_ is the total number of contacts having a distance of *j* bp, and *a* and *b* are the upper and lower bound of bin *i*. The logarithms of the 50 points {(C_1_, D_1_), … (C_50_, D_50_)} were fitted with the “smooth.spline” function in the R “stats” package (with “spar” set as 0.2). We ensured that at the end of fitting, the *C* values were monotonically decreasing with respect to the *D* values. Based on the regression model *f*(*D*), the contact probabilities with neighbors were calculated for each anchor point (segment) in the genome as

(3) $$p_{i}=\frac{f(D_{i})\cdot l_{i}}{\sum_{j=1}^{n}\;[f(D_{j})\cdot l_{j}]},$$

where (*j* = 1, 2, … n) are all upstream and downstream segments within the distance range of 2–25 kb, *l*_*i*_ is the length (bp) of segment *i*, and *D*_*i*_ is the distance (bp) between the anchor point and segment *i*.

For every anchor segment, its contact probabilities (*p*) with neighboring segments were adjusted to account for technical biases. First, we considered biases due to uneven PCR amplification and different mapping ability, which directly affected the likelihood that a Hi-C read finally appeared in our filtered reads list. For example, considering an extreme situation, in which all upstream segments of segment X are either nonmappable or are very inefficiently amplified during PCR, after filtering, segment X might appear to only interact with its downstream regions. Thus, the contact probabilities *p* of segment X with its upstream regions are actually 0, since they are not detectable under our protocol. Accordingly, the probabilities *p* with downstream regions should be doubled. Therefore, the *p*_*1*_, *p*_*2*_, …*p*_*n*_ values of interacting partners of an anchor segment were adjusted based on their “visibility,” which is in turn associated with PCR (mainly due to GC content biases) and mappability biases. We assayed these biases by analyzing genomic resequencing data of *A. thaliana*, in which the genomic DNA are fragmented randomly. Thus, prior to library amplification, all chromatin segments that we defined in this study should be equally represented in genomic resequencing data sets. After mapping, the differences of sequencing depth among chromatin segments were largely due to biases in PCR and the mapping process. We analyzed 10 genomic resequencing data sets from *A. thaliana* Col-0, with each of them having about 20 million reads (Supplemental Fig. S13; Jiang et al. 2014). From these data sets, each mapped read was assigned to a chromatin segment according to its mapping position. After normalization by segment lengths, we defined sequencing bias of a chromatin segment, *β*, as the ratio between segment coverage and the average value of all segments across the genome. We removed contacts between chromatin segments with *β* below 0.05 or above 20. For the remaining contacts, the contact probability *p*_*i*_ was rewritten as

(4) $$p_{AB}=\frac{f(D_{B})\cdot l_{B}\cdot \beta_{B}}{\sum_{j=1}^{n}\;[f(D_{j})\cdot l_{j}\cdot \beta_{j}]},$$

where *B* is an interacting partner of anchor segment A, and *β*_*B*_ is the sequencing bias of segment B.

Another technical bias was restriction cutting site density, which we reported previously on a Hi-C map normalized with 2-kb windows (Wang et al. 2015). When using small window sizes, the sequencing depth of windows with fewer cutting sites tended to be lower. In this study, we found a log-linear relationship between sequencing depth of chromatin segments and the numbers of fragment ends (Supplemental Fig. S3B). Thus, we further revised *p*_*AB*_ as

(5) $$p_{AB}=\frac{f(D_{B})\cdot f(E_{B})\cdot l_{B}\cdot \beta_{B}}{\sum_{j=1}^{n}\;[f(D_{j})\cdot f(E_{j})\cdot l_{j}\cdot \beta_{j}]},$$

where *f*(*E*_*B*_) is the regression model describing the Hi-C sequencing depth of a chromatin segment B as a function of the number of fragment ends in segment B.

For each pair of chromatin segments (segment A and segment B), we observed its contact AB and considered it as *AB* = *X* + *Y*, where *X* and *Y* had negative binomial distributions as *X* ∼ *NB*(*A*, *p*_*AB*_) and *Y* ∼ *NB*(*B*, *p*_*BA*_), describing a combination event of taking segment A and segment B as anchor points. Therefore, the probability of observing *k* reads was

(6) $$P(AB=k)=\overset{k}{\underset{i=0}{\sum }}\;[P(X=i)\cdot P(Y=k-i)],$$

which could be rewritten as

(7) $$P(AB=k)=\overset{k}{\underset{i=0}{\sum }}\;[f(i,\;A,p_{AB})\cdot f(i,\;B,p_{BA})],$$

where *A* and *B* are background reads on segment A and B excluding AB reads, *p*_*AB*_ and *p*_*BA*_ are adjusted contact probability described above, and

(8) $$f(k,r,p)=\left(\begin{array}{c} k+r-1\\ k \end{array}\right)\cdot p^{k}\cdot {\left(1-p\right)}^{r}$$

is the probability mass function of the negative binomial distribution. From this model, we calculated the *p* value of observing *k* reads as

(9) $$p=1-\overset{k-1}{\underset{j=0}{\sum }}\;P(AB=j).$$

Lastly, the parameters of background reads of segment A and B were estimated as

(10) $$\hat{A}=\overset{n}{\underset{i=1}{\sum }}\;C_{Ai}\cdot r_{i}$$

and

(11) $$r_{i}=\left\{ \begin{array}{c} 0.5,\;\mathrm{if}\;\mathrm{the}\;\mathrm{denominator}\;\mathrm{is}\;0\\ \frac{A_{\mathrm{total}}-C_{Ai}\;}{(A_{\mathrm{total}}-C_{Ai})+(i_{\mathrm{total}}-C_{Ai})\;},\;\mathrm{otherwise} \end{array}\right.$$

where *C*_*Ai*_ is the read count between segment A and its interacting partner *i*, and *A*_*total*_ and *i*_*total*_ the sequencing depth of segment A and segment *i*, respectively. By scanning through all interacting partners of segment A, we used the estimated *Â* as the total number of failed trials (considering segment A as the anchor point) in our negative binomial distribution model. At the end of the *p* value calculation, multiple testing correction was performed with the Benjamini-Hochberg method to obtain *q* values. Among all contacts observed, we selected those with *q* values less than 0.01 for downstream association analysis. Association analysis pipelines regarding promoter-promoter interactions, gene body to gene body interactions, and gene loops are illustrated in Supplemental Figure 14.

### Calling gene loops

For each annotated gene, we used its TSS, as well as bases at 400 and 800 bp downstream from the TSS (if they reside inside the gene body) as focal points. After each focal point was assigned to the corresponding chromatin segment, we looked for all interacting partners of this segment in our data set with *q* values below 0.01. We only retained interacting partners within the search radius, which was the distance from the focal point to the TTS. In this way, a gene was identified as having gene loop conformation if its focal point(s) had at least one interacting partner located at the 3′ downstream end with respect to the direction of transcription (Fig. 5C). Note that we only considered chromatin loops with distances between 2 and 25 kb and did not attempt to call gene loops for short genes. This left 14,672 genes as candidates for calling gene loops.

### ChIP-seq library preparation and analysis

Seedlings were fixed under vacuum for 30 min with 1% formaldehyde in MC buffer (10 mM potassium phosphate, pH 7.0, 50 mM NaCl, 0.1 M sucrose) at room temperature. After fixation, seedlings were incubated at room temperature for 5 min under vacuum in MC buffer with 0.15 M glycine. Nuclei were isolated as described (Wang et al. 2015), and nuclei from 1 g fixed material were used for each round of ChIP. Isolated nuclei were resuspended in 1 mL sonication buffer (10 mM potassium phosphate, pH 7.0, 0.1 mM NaCl, 0.5% sarkosyl, 10 mM EDTA), and chromatin was sheared by sonication with a Covaris S220 instrument to achieve average fragment size around 300 bp. The sonicated sample was mixed with 100 µL 10% Triton X-100, and 50 µL of this was saved as input sample. The rest of the sheared chromatin was mixed with an equal volume of IP buffer (50 mM Hepes, pH 7.5, 150 mM NaCl, 5mM MgCl_2_, 10 µM ZnSO_4_, 1% Triton X-100, 0.05% SDS) and incubated with anti-Pol II antibody (Abcam ab5408), or equally divided and incubated with anti-H3 (Abcam ab1791) and anti-H3K27me3 antibodies (Millipore, 07-449). After overnight incubation at 4°C, 10 µL Protein A/G magnetic beads (Pierce) were added and incubated at 4°C for 2 h. The beads were washed at 4°C as follows: 3× with IP buffer, 1× with IP buffer having 500 mM NaCl, and 1× with LiCl buffer (0.25 M LiCl, 1% NP-40, 1% deoxycholate, 1mM EDTA, 10 mM Tris pH 8.0) for 5 min each. Chromatin retained on beads were incubated in 200 µL elution buffer (50 mM Tris, pH 8.0, 200 mM NaCl, 1% SDS, 10 mM EDTA) at 65°C for 6 h, followed by Proteinase K treatment at 37°C for 1 h. DNA was extracted with a standard phenol-chloroform method, and all subsequent end repairing, A-tailing, adaptor ligation, and library amplification steps were done following a standard protocol (Illumina). The final library was sequenced on an Illumina HiSeq 2000 instrument with 1×101-bp reads.

Reads were aligned against the *A. thaliana* reference genome (TAIR10) using Bowtie 2 v2.2.4 (Langmead and Salzberg 2012) with mapping parameters as “-R 5 -N 0 -L 20 -i S,1,0.50.” The mapped reads were analyzed by MACS2 v2.1.0.20140616 (Zhang et al. 2008). The “--broad” flag was on for both Pol II and H3K27me3 peak calling, with reads from the input or anti-H3 sample used as controls, and default settings were used for the rest parameters.

## Data access

Hi-C and ChIP-seq short read data from this study have been submitted to the NCBI Sequence Read Archive (SRA; http://www.ncbi.nlm.nih.gov/sra/) under accession number SRP064711.
